# Cardiac MRI in infarct-like myocarditis: transmural extension of late gadolinium enhancement is associated with worse outcomes

**DOI:** 10.1186/s13244-024-01832-3

**Published:** 2024-10-11

**Authors:** Alexander Isaak, Johannes Wirtz, Dmitrij Kravchenko, Narine Mesropyan, Leon M. Bischoff, Simon Bienert, Leonie Weinhold, Claus C. Pieper, Ulrike Attenberger, Can Öztürk, Sebastian Zimmer, Daniel Kuetting, Julian A. Luetkens

**Affiliations:** 1 Clinic for Diagnostic and Interventional Radiology, University Hospital Bonn, Bonn, Germany; 2 Quantitative Imaging Lab Bonn (QILaB), University Hospital Bonn, Bonn, Germany; 3 Institute of Medical Biometry, Informatics, and Epidemiology, University Hospital Bonn, Bonn, Germany; 4 Heart Center, Department of Internal Medicine II, University Hospital Bonn, Bonn, Germany

**Keywords:** Heart, Myocarditis, Magnetic resonance imaging, Outcome assessment, Major adverse cardiac events

## Abstract

**Objectives:**

To assess the prognostic value of cardiac MRI (CMR) parameters for the occurrence of major adverse cardiac events (MACE) in patients with infarct-like myocarditis.

**Methods:**

In this retrospective single-center study, patients with CMR-confirmed acute myocarditis with infarct-like presentation were identified (2007–2020). Functional and structural parameters were analyzed including late gadolinium enhancement (LGE). The primary endpoint was the occurrence of MACE up to 5 years after discharge.

**Results:**

In total, 130 patients (mean age, 40 ± 19 years; 97 men, 75%) with infarct-like myocarditis were included. CMR was conducted a median of 3 days (interquartile range [IQR], 1–5) after symptom onset. MACE occurred in 18/130 patients (14%) during a median follow-up of 19.3 months (IQR, 4.5–53). The median extent of LGE was 7% (IQR, 4–10). LGE affected the subepicardium in 111/130 patients (85%), the midwall in 45/130 patients (35%), and both the subepicardium and midwall in 27/130 patients (21%). Transmural extension of non-ischemic LGE lesions was observed in 15/130 patients (12%) and septal LGE in 42/130 patients (32%). In univariable Cox regression analysis, a significant association was found between the occurrence of MACE and both, quantified LGE extent and transmural LGE pattern. In multivariable analysis, transmural extension of LGE was an independent predictor for MACE (hazard ratio, 6.34; 95% confidence interval: 2.29–17.49; *p* < 0.001). Patients with the transmural extension of LGE had a shorter event-free time on Kaplan–Meier analysis (log-rank *p* < 0.001).

**Conclusions:**

MACE occurred in 14% of patients with infarct-like myocarditis during follow-up. A transmural extension of non-ischemic LGE was associated with a worse long-term prognosis.

**Critical relevance statement:**

CMR-based assessment of transmural extension of non-ischemic LGE holds the potential to serve as an easily assessable marker for risk stratification in patients with infarct-like myocarditis.

**Key Points:**

- The prognostic value of CMR was studied in patients with infarct-like myocarditis.
- The extent of LGE and transmural extension were linked to adverse cardiac events.
- Transmural non-ischemic LGE can serve as an easily assessable prognostic marker.

**Graphical Abstract:**

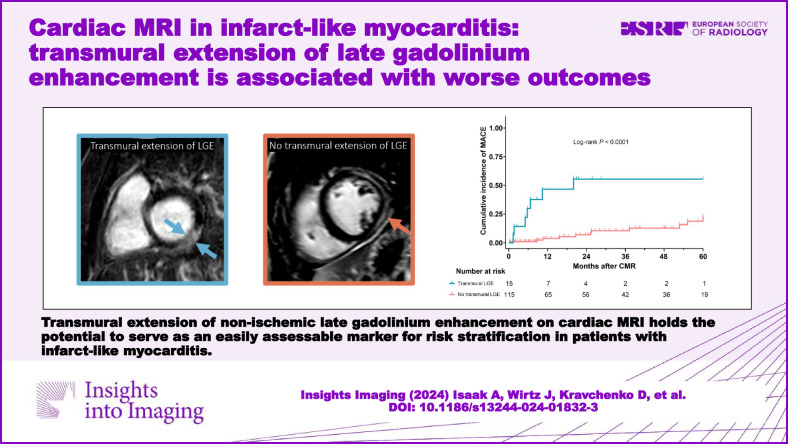

## Introduction

Acute myocarditis poses a diagnostic challenge due to its heterogeneous clinical presentation, ranging from a subclinical to a potentially fulminant course of disease. A so-called infarct-like presentation of acute myocarditis, characterized by chest pain and ST-segment abnormalities on electrocardiogram and/or troponin elevation, has been described in up to 46% of patients [1]. Infarct-like myocarditis can mimic acute coronary syndrome and may lead to delayed or inappropriate treatment. Among patients referred for cardiac MRI (CMR) in the setting of suspected myocardial infarction and unobstructed coronary arteries, acute myocarditis emerged as one of the prevailing diagnoses, comprising approximately 33% of cases [2].

The majority of patients with acute myocarditis have preserved left ventricular (LV) ejection fraction and a predominantly uncomplicated clinical course [1]. Previous outcome studies demonstrated that clinical and imaging parameters such as late gadolinium enhancement (LGE), myocardial edema, ejection fraction, and age are related to the long-term prognosis of patients with myocarditis [3–5]. However, there are conflicting results concerning the long-term outcome of patients with infarct-like myocarditis. While some CMR studies report a predominantly favorable prognosis compared to other clinical presentations of myocarditis like non-infarct-like myocarditis [6, 7] others have found a nonnegligible rate of major adverse cardiovascular events (MACE) [8]. Currently, the prognostic role of CMR parameters in infarct-like myocarditis has not yet been sufficiently elucidated.

The aim of this study was to assess the prognostic value of CMR parameters regarding the occurrence of MACE among patients with infarct-like myocarditis.

## Materials and methods

### Study population

This was a retrospective, observational single-center study that was conducted at a tertiary care center. From August 2007 to February 2020, patients with CMR-confirmed acute myocarditis were identified in the radiological information system [9]. Only patients with an infarct-like presentation of myocarditis were included in the final study population, defined as chest pain with either ST-segment abnormalities or troponin level elevation (or with both ST-segment abnormalities and troponin level elevation). Patients with concomitant myocarditis and obstructive coronary heart disease (e.g., coronary stenosis on coronary angiogram > 50% or myocardial infarction on CMR) were not included. Patients with non-obstructive coronary artery disease (defined as coronary stenosis < 50%) on coronary angiogram and clinically and CMR-confirmed acute myocarditis were included. The study complies with the principles of the Helsinki Declaration. The local institutional review board approved this study and waived informed consent due to the retrospective study design.

### Cardiac magnetic resonance

CMR was performed using a 1.5-T magnetic resonance system (1.5 Ingenia, Philips Healthcare). A 16-channel body array coil with a digital interface was employed to facilitate signal reception. For functional analysis, electrocardiogram-gated steady-state free precession cine imaging was performed in four-, three-, two-chamber, and short-axis views. For the assessment of myocardial edema, T2-weighted short-tau inversion-recovery imaging in short-axis and transversal views was performed. LGE imaging using segmented inversion-recovery gradient-echo sequence in four-, three-, two-chamber, axial, and short-axis views were acquired starting 8–10 min after contrast injection (single bolus of 0.2 mmol/kg body weight of gadobutrol; Gadovist, Bayer Healthcare) for the detection of myocardial hyperenhancement (necrosis or fibrosis). Parametric mapping sequences were available in a subgroup of patients; T1 mapping based on a 3(3)3(3)5 modified Look-Locker inversion recovery [MOLLI] acquisition scheme and T2 mapping based on a 6-echo gradient spin echo [GraSE] sequence as previously described [10, 11].

### Cardiac magnetic resonance analysis

CMR studies were evaluated using dedicated software (IntelliSpace Portal Version 12, Philips Medical System). Analyses were performed in consensus agreement between two board-certified cardiovascular radiologists (A.I., 7 years of experience in CMR; J.A.L. 12 years of experience in CMR) according to current recommendations [12]. All functional and structural CMR analyses were performed by the first radiologist and results were supervised by the second radiologist. The readers were blinded to clinical results and outcomes. Functional parameters (e.g., LV end-diastolic volume, LV ejection fraction) were calculated by manually drawing contours in the end-diastolic and end-systolic steady-state free precession cine images in short-axis view (including papillary muscles in the LV cavity). Regional wall motion abnormalities were visually assessed. Assessment of myocardial edema was done by visual and semi-quantitative approach (T2 signal intensity ratio) [13]. LGE was qualitatively assessed according to its presence (17-segment model) and its distribution (subendocardial, mid-wall, subepicardial, transmural, pericardial). A combined pattern of subepicardial and mid-wall LGE was defined as present if both the subepicardial and the mid-wall were affected in at least one segment. A transmural extension of non-ischemic LGE (primary subepicardial pattern) was defined as complete LV wall thickness occupancy involving all three myocardial layers in at least one segment. Septal LGE was defined as the involvement of at least one septal segment according to the 17-segment model (segments 2, 3, 8, 9, and 14). The extent of LGE (LGE percentage) was quantitatively measured using the full width half maximum technique as previously described [11, 12, 14]; epicardial and endocardial LV contours were manually drawn on all LGE images in short axis view and LGE mass was quantified based on regions with signal intensity above 50% of the maximum. Artifacts were manually removed. If available, global myocardial T1 and T2 relaxation time, and hematocrit corrected extracellular volume fraction values were calculated from three slices in short axis views (basal, midventricular, and apical) according to current recommendations; and center-specific reference values were applied as previously described [15–17]. The presence of a pericardial effusion (> 10 mm) and a pleural effusion (> 10 mm) was assessed.

### Outcome assessment

Follow-up data were collected by reviewing in-house and outpatient medical records. MACE was defined as a composite including cardiovascular death (death directly related to heart failure, myocardial infarction, arrhythmia, or cardiovascular disease), re-hospitalization due to new onset of acute symptoms (e.g., recurrence of myocarditis), or heart failure symptoms, and implantation of a pacemaker or implantable cardioverter-defibrillator. The primary endpoint was the occurrence of MACE up to 5 years after discharge.

### Statistical analysis

Prism (version 8.4.3; GraphPad Software Inc.) and SPSS Statistics (version 26; SPSS Inc., IBM) were used for statistical analysis. Normal distribution of the data was confirmed by visual inspection supplemented by the Shapiro–Wilk test. Continuous data variables were expressed as means ± standard deviation and discrete data variables as medians with interquartile range (IQR). Categorical variables were presented as total frequencies, with corresponding percentages in parentheses. The association between clinical and imaging parameters and MACE was tested using an univariable Cox regression analysis. Covariates with *p* < 0.05 in the univariable analysis were included in a multivariable model to better ascertain the impact of these variables. Event rates were examined using a Kaplan–Meier analysis with stratification according to the presence of transmural LGE. A log-rank test was utilized to compare the survival curve. The statistical significance threshold was set at *p* < 0.05.

## Results

**Fig. 1:**
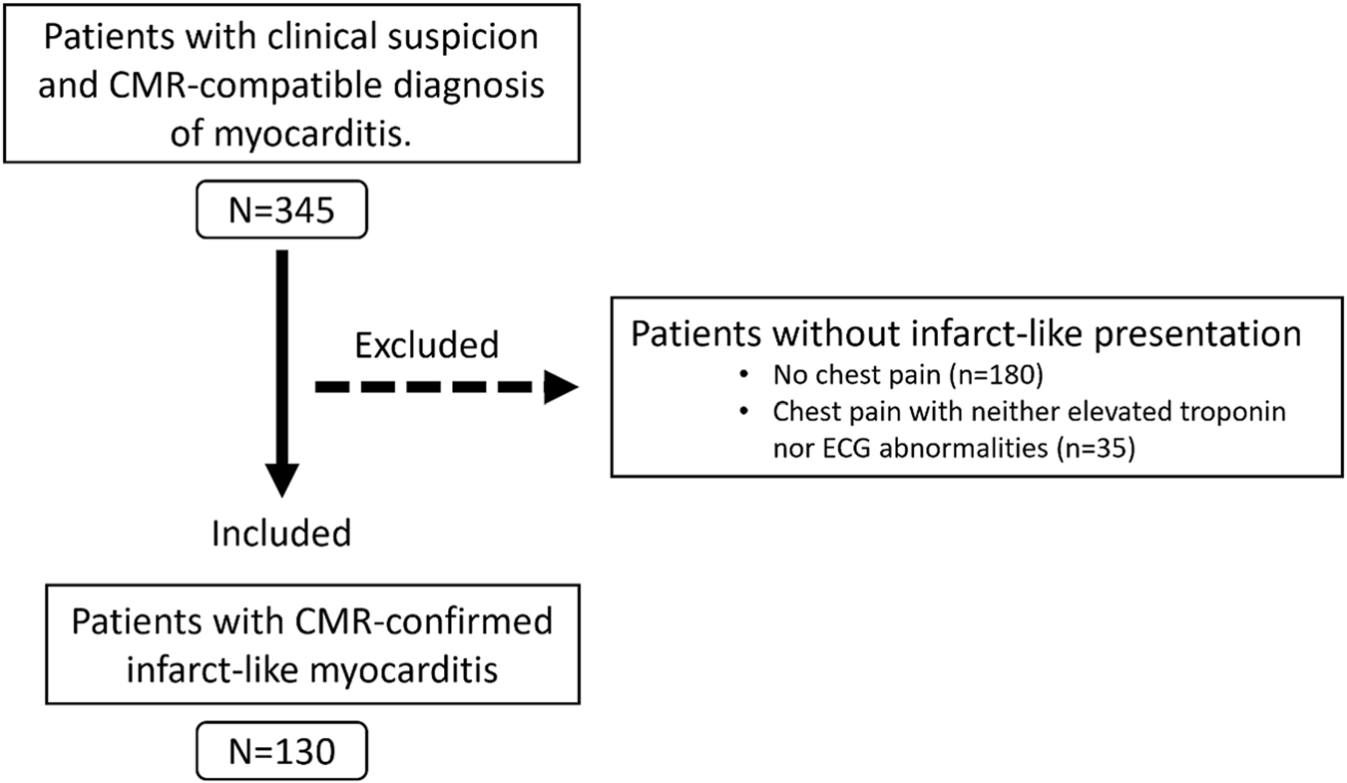
Study flow chart. CMR, cardiac MRI

Of 345 retrospectively identified patients with clinically suspected and CMR-compatible diagnosis of myocarditis, 130 patients had an infarct-like clinical presentation (mean age, 40 ± 19 years; 97 men, 75%) and were further analyzed. A study flow diagram is presented in (Fig. 1).

### Clinical characteristics

Demographic and clinical data are shown in Table 1. According to the study inclusion criteria all patients had chest pain at initial clinical presentation, and most patients had elevated troponin levels (125/130, 96%). Coronary angiography was performed in 93/130 patients (72%) with exclusion of obstructive coronary artery disease in all patients (93/93, 100%) and diagnosis of non-obstructive coronary artery disease (coronary stenosis < 50%) in 18/93 patients (19%); none of these patients had ischemic scars on CMR or other clinical results indicating significant ischemic heart disease resulting in clinically and CMR-based diagnosis of acute myocarditis in all cases. Associated infectious disease was present in 84/130 patients (65%). Eighteen of one hundred thirty patients (14%) were admitted to intermediate care or intensive care units for treatment or monitoring.

**Table 1:** Characteristics of patients with infarct-like myocarditis

Parameters	Total cohort, (*n* = 130)
General parameter	
Age, (years)	40 ± 19
Sex, (male)	97 (75%)
Body mass index, (kg/m²)	26 ± 5
Clinical presentation on admission	
Chest pain	100 (100%)
Associated infectious disease	84 (65%)
ST segment abnormality	70 (54%)
ICU or IMC stay	18 (14%)
Coronary angiography performed	93 (72%)
Obstructive coronary artery disease	0/93 (0%)
Non-obstructive coronary artery disease	18/93 (19%)
Endomyocardial biopsy performed	10/130 (8%)
Laboratory data	
Elevated troponin T/I^a^	125 (96%)
Troponin T, (ng/L)^a^	653 (241–1022)
Troponin I, (ng/L)^a^	4.00 (0.62–9.93)
NT pro-BNP, (pg/mL)^a^	116 (44–1060)
CK-MB mass, (ng/mL)^a^	14.5 (2.8–34.3)
White blood cell count, (10³/µL)	9.9 (7.6–12.4)
C-reactive protein, (mg/L)	56 (11–102)
Cardiac magnetic resonance	
Pericardial effusion	18 (14%)
Pleural effusion	13 (10%)
Heart rate, (bpm)	69 ± 12
LV ejection fraction, (%)	56 ± 12
LV end-diastolic volume index, (mL/m^2^)	74 ± 21
Interventricular septal thickness, (mm)	9.6 ± 1.8
Visible focal myocardial edema, (T2 STIR)	130 (100%)
T2 signal intensity ratio, (T2 STIR)	2.4 ± 0.5
T1 relaxation time, native, (ms)^b^	1033 ± 67
T2 relaxation time, (ms)^b^	60 ± 7
Extracellular volume fraction, (%)^b^	29 ± 4
LGE presence	130 (100%)
Subendocardial LGE	0 (0%)
Subepicardial LGE	111 (85%)
Midwall LGE	45 (35%)
Subepicardial and mid wall LGE	27 (21%)
Transmural LGE	15 (12%)
Septal LGE	42 (32%)
Pericardial LGE	24 (18%)
LGE, quantified, (%)	7 (4–10)
No. of segments with LGE	3 (2–5)

Numbers are given as mean ± standard deviation, median (IQR), and frequency (percentage)

*NT* *pro-BNP* N-terminal pro-B-type natriuretic peptide, *CK-MB mass* creatin-kinase muscle-brain type, *ICU* intensive care unit, *IMC* intermediate care, *STIR* short tau inversion recovery, *LGE* late gadolinium enhancement

^a^ Laboratory data was available as follows: NT pro-BNP was available in 9/130 patients (7%), CK MB was available in 116/130 patients (89%), troponin T was available in 42/130 patients (27%), troponin I was available in 95/130 patients (73%). Elevated troponin was defined as troponin I ≥ 0.05 ng/L and troponin T ≥ 14 ng/L

^b^ T1/T2 mapping and ECV were available in 42/130 patients (32%)

### CMR characteristics

**Fig. 2:**
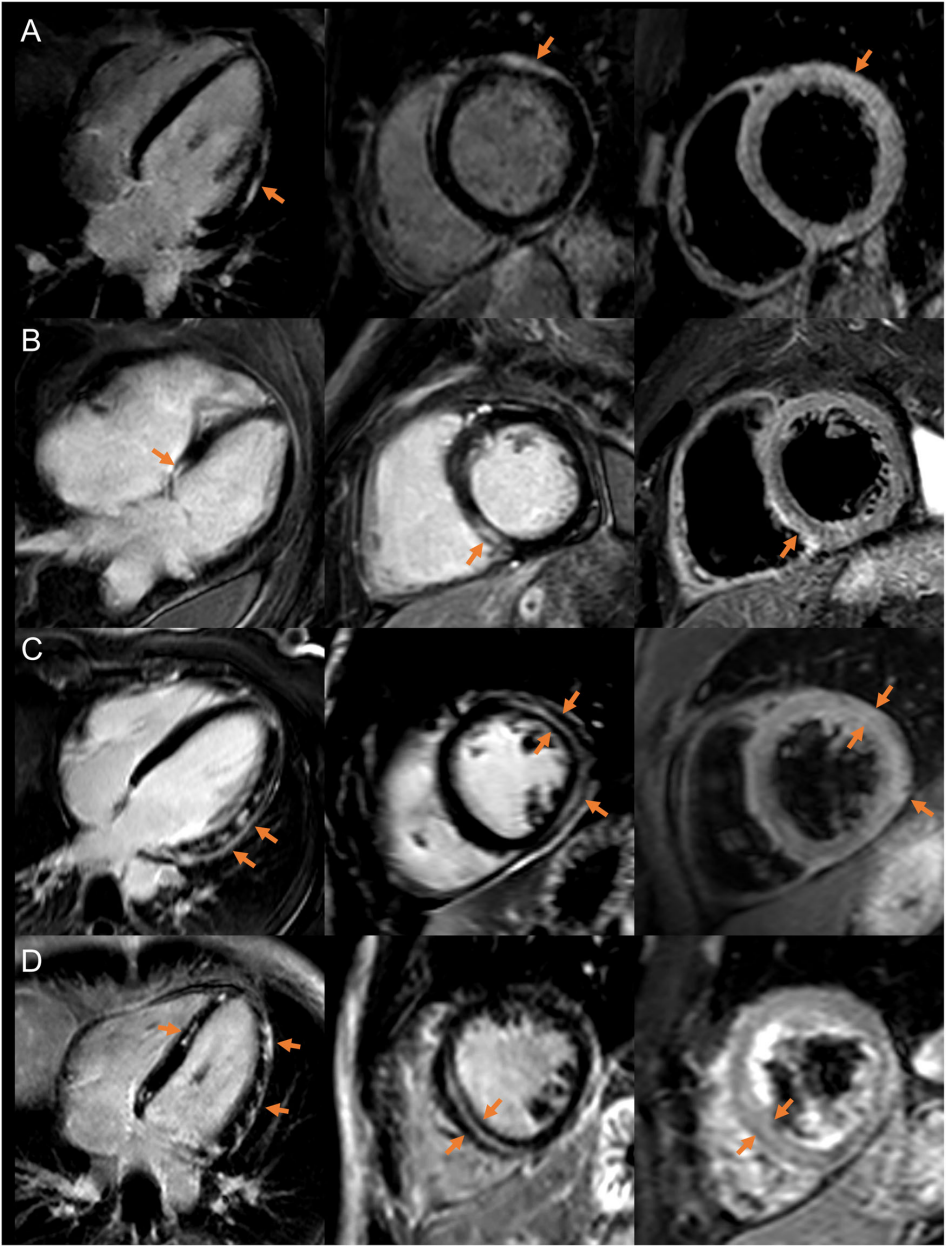
Cardiac magnetic resonance imaging examples of patients with different LGE patterns: (**A**) subepicardial, (**B**, **D**) midwall with septal involvement, and (**C**) subepicardial and midwall. Corresponding LGE lesions with focal edema are marked with orange arrows, respectively. Left column: LGE in four-chamber view; middle column: LGE in short axis view; and right column: fat-suppressed T2 sequence in short axis view

**Fig. 3:**
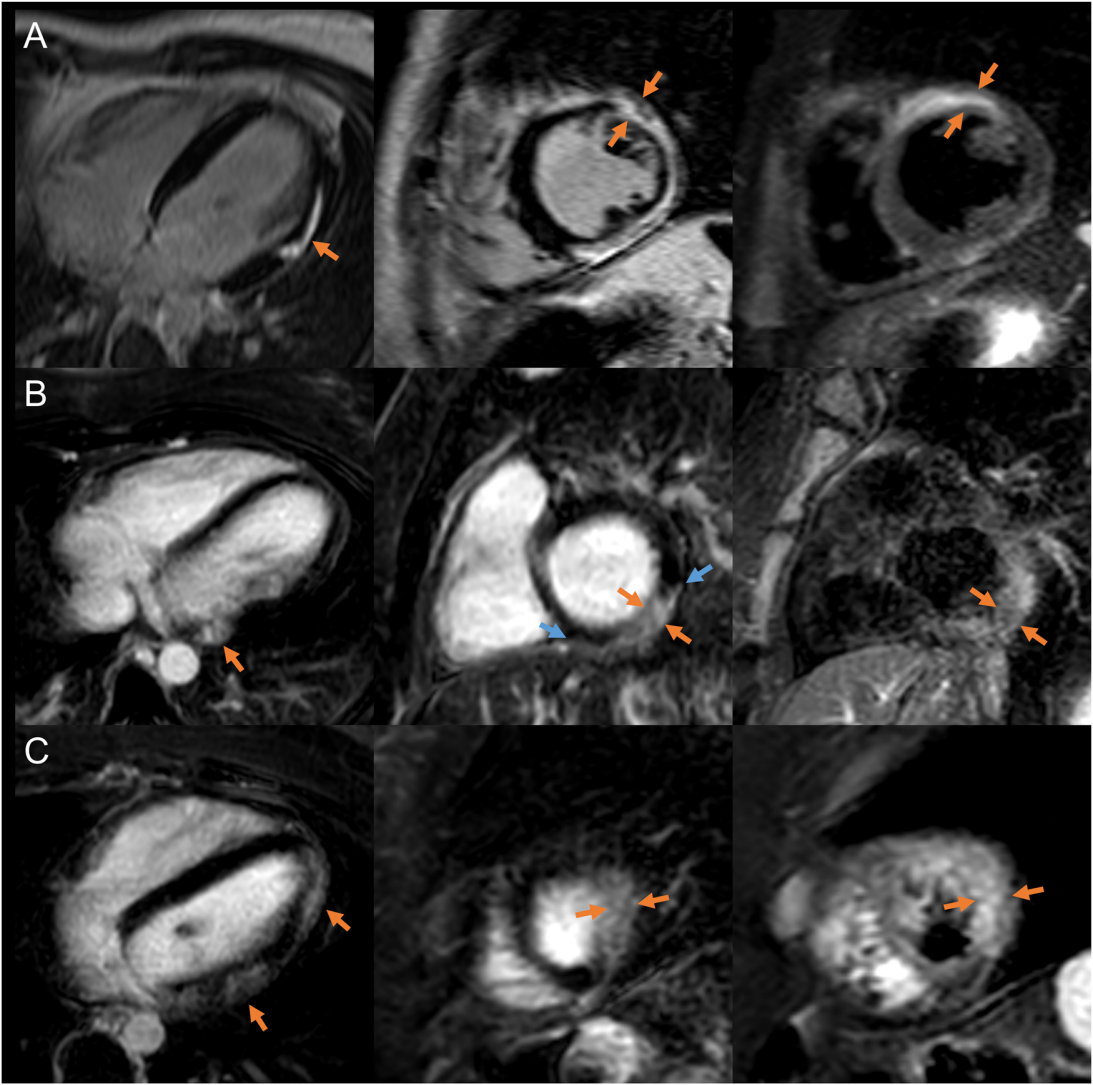
Clinical examples of LGE with transmural extension and corresponding edema (orange arrows). **A** Nineteen-year-old male patient presented with chest pain, elevated troponin, and ST-segment abnormalities but no associated infectious disease. Diagnosis of acute lymphocytic myocarditis was confirmed by CMR and endomyocardial biopsy. The patient experienced a recurrence of acute myocarditis approximately one year after the initial diagnosis. **B** Thirty-three-year-old female patient with flu-like symptoms and acute chest pain and troponin elevation without abnormalities on electrocardiogram. Clinical diagnosis of acute myocarditis was confirmed by CMR; the middle image shows LGE with a non-ischemic pattern (laterally the subepicardium is involved; blue arrows) and transmural extension. **C** Thirty-one-year-old female patient with previous tonsillitis and clinically infarct-like presentation. Acute myocarditis was diagnosed based on CMR. On clinical follow-up, the patient developed a grade 3 intermittent atrioventricular block. Left column: LGE in four-chamber (**A**) or axial view (**B**, **C**); middle column: LGE in short axis view; and right column: fat-suppressed T2 sequence in short axis view

CMR data results are given in Table 1. CMR has conducted a median of 3 days (IQR, 1–5) after symptom onset. Most patients (121/130, 93%) had a preserved LV ejection fraction (≥ 50%). All patients had non-ischemic LGE lesions with associated myocardial edema. No concomitant ischemic scar was found in the cohort. LGE lesions involved the subepicardium in 111/130 patients (85%), the midwall in 45/130 patients (35%), and both in 27/130 patients (21%) (Fig. 2). Transmural extension of non-ischemic LGE was present in 15/130 patients (12%) (Fig. 3). Septal LGE was present in 42/130 patients (32%). LGE affected a median of 3 segments (IQR, 2–5). The median extent of LGE was 7% (IQR, 4–10). 16/130 patients (12%) had an LGE extent > 15%. A pericardial effusion was present in 18/130 patients (14%) and a pleural effusion in 13/130 patients (10%).

### Outcome

**Fig. 4:**
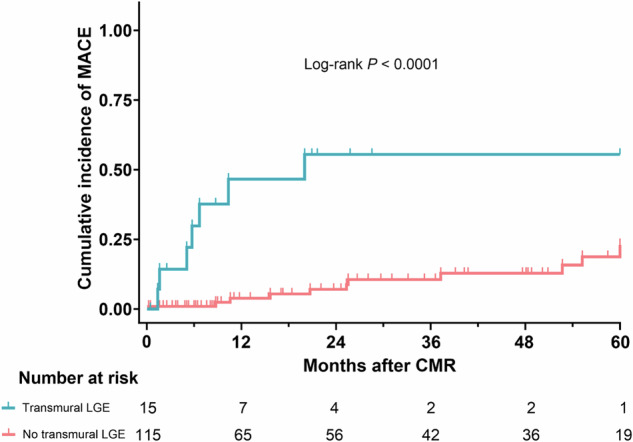
Kaplan–Meier curve shows the difference in cumulative MACE depending on the presence of transmural LGE

The median follow-up was 19.3 months (IQR, 4.5–53). MACE occurred in 18/130 patients (14%) during follow-up. The median time between CMR and MACE was 11 months (IQR, 6–28). All events are listed in Table 2. In univariable Cox regression analysis, an association was found between the occurrence of MACE and both, the quantified extent of LGE and the transmural extension of LGE (Table 3). No significant association was found between MACE and different general and laboratory parameters (e.g., age, diagnosis of non-obstructive coronary artery disease, intensive care unit stay, and troponin), as well as functional CMR parameters in univariable analysis (Table 3). In multivariable Cox regression analysis, the transmural pattern of LGE was an independent predictor for MACE (hazard ratio, 6.34; 95% confidence interval [CI]: 2.29–17.49; *p* < 0.001; Table 4). Kaplan–Meier analysis showed that patients with a transmural extension of LGE had a lower probability of remaining event-free than patients without transmural LGE (median event-free time approximately 22 months vs more than 60 months; log-rank *p* < 0.001; see Fig. 4).

**Table 2:** Major adverse cardiac events during follow-up

Clinical events, (*n* = 18)	Patients, (*n* = 130)
Re-hospitalization due to new onset of acute cardiac symptoms	7 (5.4%)
Re-hospitalization due to new onset of heart failure symptoms	7 (5.4%)
Pacemaker or implantable cardioverter-defibrillator implantation	2 (1.5%)
Cardiovascular death	2 (1.5%)

**Table 3:** Univariable Cox regression analyses for the prediction of MACE

	Univariable analysis	
Parameter	Hazard ratio	*p* value
Age (per year)	1.01 (0.98–1.03)	0.51
Sex (male)	0.97 (0.35–2.73)	0.96
Body mass index (per kg/m²)	0.93 (0.85–1.02)	0.14
Heart rate (per bpm)	1.04 (1.00–1.08)	0.06
Non-obstructive coronary artery disease	3.30 (0.79–13.88)	0.10
ICU or IMC stay	0.67 (0.15–2.93)	0.60
White blood cell count (per 10³/µL)	0.97 (0.86–1.09)	0.56
Troponin I level (per ng/L)	1.02 (0.98–1.05)	0.34
Pleural effusion (> 10 mm)	2.97 (0.98–9.04)	0.055
Pericardial effusion (> 10 mm)	1.02 (0.23–4.45)	0.98
LV ejection fraction (per %)	0.98 (0.94–1.01)	0.18
T2 signal intensity ratio	0.62 (0.20–1.95)	0.41
LGE, quantified (per %)	1.08 (1.02–1.15)	0.009
No. of segments with LGE	1.04 (0.89–1.22)	0.63
Subepicardial LGE	1.12 (0.26–4.89)	0.88
Midwall LGE	0.97 (0.36–2.58)	0.94
Subepicardial and mid wall LGE	1.31 (0.47–3.68)	0.61
Septal LGE	0.79 (0.30–2.03)	0.62
Transmural extension of LGE	7.66 (2.89–20.32)	< 0.001

Hazard ratios are given with 95% CIs

*ICU* intensive care unit, *IMC* intermediate care unit, *LGE* late gadolinium enhancement

**Table 4:** Multivariable Cox regression analysis for the prediction of MACE

	Multivariable analysis	
Parameter	Hazard ratio	*p* value
LGE (per %)	1.06 (0.10–1.13)	0.059
Transmural extension of LGE	6.34 (2.29–17.49)	< 0.001

Hazard ratios are given with 95% CIs

## Discussion

Our main study results show that 14% of patients with infarct-like myocarditis experienced MACE during a median follow-up period of 19.3 months. Furthermore, the presence of a transmural extension of non-ischemic LGE was associated with a worse long-term prognosis (hazard ratio, 6.34; 95% CI: 2.29–17.49; *p* < 0.001). These findings underscore the important role of LGE assessment for risk stratification in infarct-like myocarditis.

The infarct-like type represents a relatively common clinical manifestation of acute myocarditis that is mainly defined by chest pain, ST-segment abnormalities on the electrocardiogram, and elevated troponin levels [1]. Functional cardiac parameters are predominantly preserved or only slightly impaired, as indicated also in the presented study cohort. Although the definition of infarct-like myocarditis varies among studies, the prevalence of infarct-like myocarditis in our study (37.7%) is broadly in line with previous studies of Francone et al (36.8%) and Capasso et al (35.9%), but is lower than in the studies of Chopra et al (54.5%) and Lurz et al (52.8%) [8, 18–20]. CMR has shown high diagnostic performance in acute myocarditis with an infarct-like presentation in a histopathological validation study [19]. However, the prognostic role of CMR in myocarditis with an infarct-like pattern remains poorly studied, with some conflicting study results having been reported in the past.

We found a relatively favorable long-term prognosis during a follow-up period of up to 5 years (median: 19.3 months) with an incidence of MACE of approximately 14%. Previous follow-up studies of patients with infarct-like myocarditis showed a predominately benign disease course. In the study by Duršpek et al, infarct-like myocarditis showed a favorable long-term prognosis without the development of chronic myocarditis or recurrence of the disease within the 1 year follow-up period, even when physical activity was started 1 month after hospital discharge [6]. Another clinical and CMR imaging follow-up study on infarct-like myocarditis by Faletti et al reported a benign prognosis without any occurrence of MACE during a 6-month follow-up interval [7]. Compared to the present cohort, lower cardiac event rates were reported by the study of Aquaro et al with approximately 8% (events: 29/374; median follow-up: 1572 days; cohort almost completely composed of patients with infarct-like myocarditis) and by Sanguineti et al with approximately 10.8% (events: 22/203; mean follow-up: 18.9 ± 8.2 months; cohort included 70% of patients with infarct-like myocarditis) [21, 22]. In contrast, a much higher MACE rate of approximately 29% was reported in the study by Chopra et al (events: 14/48; median follow-up: 16 months), and the infarct-like pattern itself was associated with MACE (HR 2.4 [1.01–5.80], log-rank *p* = 0.04) [8]; however, the number of patients for outcome analysis was rather small in that study.

Our study results demonstrated an association between the total extent of LGE (measured as LGE percentage) and MACE using univariable analysis. A higher incidence of MACE among patients with higher LGE extent is widely consistent with several previous myocarditis studies [4, 22–24], including the study of Chopra et al that was also focused on infarct-like myocarditis [8]. However, the study by Sanguineti et al found no significant association with outcome and LGE, but with altered LV ejection fraction [21]. The somewhat heterogeneous results between these studies might be explained by the different methods used to quantify LGE. LGE is a marker of myocardial injury, and its extent mirrors the degree of myocardial necrosis in myocarditis. Therefore, it would be plausible that a greater extent of LGE could reflect a more widespread disease process that may influence the likelihood of MACE.

Previous studies have postulated that the location and pattern of LGE may predict outcomes in myocarditis. Aquaro et al reported that mid-wall LGE affecting the anteroseptal segments was associated with a worse prognosis than other patterns [22]. In the study by Gräni et al a septal and mid-wall pattern of LGE showed the strongest associations with MACE [4]. A potential hypothesis for these findings is that inflammatory involvement of the conduction system may trigger arrhythmic events (e.g., inflammation caused by the human herpesvirus 6 that also affects the nervous system) [22]. A septal involvement of LGE was neither significantly associated with MACE in the study by Imazio et al nor in our study. The generally low number of MACE and the specific cohort focused solely on infarct-like myocarditis could be potential explanations.

Our study results suggest that a full transmural extension of non-ischemic LGE might be a new and important predictor of MACE in patients with infarct-like myocarditis. These findings demonstrate that not just the total quantified extent of LGE, but also the transmural extension of LGE involving the subendocardial layers might carry prognostic relevance. Interestingly, we found no significant association between the occurrence of MACE and the total number of LGE segments, but did observe an association with MACE and quantified LGE (LGE percentage). This might also indicate that the transmural extension of LGE within a segment might be more associated with long-term prognosis than the extension across multiple segments. Although the mechanistic pathways linking different LGE patterns to MACE require further elucidation in different types of myocarditis, it is in principle conceivable that transmural extension of LGE (indicative of full-thickness myocardial necrosis) may represent a more severe or atypical phenotype of myocarditis that is associated with a greater risk of MACE. Interestingly, transmural extension of LGE is also known to be associated with advanced disease forms and worse outcomes in patients with other non-ischemic myocardial diseases like cardiac amyloid, cardiac involvement in muscular dystrophy, and non-ischemic dilated cardiomyopathy [25–27]. However, larger confirmatory studies are needed in patients with myocarditis and other non-ischemic cardiomyopathies that also cover specific LGE patterns like transmural or subendocardial involvement. For example, Li et al showed that patients with biopsy-proven myocarditis and subendocardial involvement on LGE had larger LGE extent, higher probability of giant cell myocarditis, and more MACE than patients without subendocardial involvement [28]. Understanding the long-term outcomes and prognostic factors associated with infarct-like myocarditis can help clinicians stratify risk, optimize patient care, and adapt treatment strategies to prevent MACE. There is a need for further research into new biomarkers to assess the prognosis of acute myocarditis.

Our study has some limitations. The retrospective and single-center nature of our study limits the generalizability of our results. Future multi-center studies would help validate these results across more diverse populations. The sample size is moderate and the observational design of our study allows for the identification of associations but cannot establish causality. Our study specifically focused on infarct-like myocarditis, and therefore our findings may not apply to other clinical presentations of myocarditis. The timing of CMR may influence the size and extent of inflammatory LGE lesions; in this cohort, CMR was performed relatively early after symptom onset, and application of the outcome parameter to patients with late CMR may be limited. Lastly, the lack of comprehensive pathologic confirmation may have led to a potential inclusion of false-positive cases of myocarditis.

In conclusion, our study highlights the prognostic importance of LGE assessment in patients with infarct-like myocarditis. Besides the overall LGE extent, transmural extension of non-ischemic LGE emerged as an independent predictor of MACE in this patient population and could represent an additional, visually easy-to-assess biomarker for risk stratification. These results emphasize the central role of CMR in patients with infarct-like myocarditis and highlight its usefulness not only for the diagnosis of myocarditis but also for its prognosis. Future prospective, multicenter studies are warranted to validate our findings and to better understand the pathophysiologic mechanisms linking LGE parameters with clinical outcomes in different types of myocarditis including the infarct-like type.

## Data Availability

Data generated or analyzed during the study are available from the corresponding author by request.

## Abbreviations

CMR: Cardiac MRI
IQR: Interquartile range
LGE: Late gadolinium enhancement
LV: Left ventricular
MACE: Major adverse cardiovascular events
